# Accurate determination of marker location within whole-brain microscopy images

**DOI:** 10.1038/s41598-021-04676-9

**Published:** 2022-01-18

**Authors:** Adam L. Tyson, Mateo Vélez-Fort, Charly V. Rousseau, Lee Cossell, Chryssanthi Tsitoura, Stephen C. Lenzi, Horst A. Obenhaus, Federico Claudi, Tiago Branco, Troy W. Margrie

**Affiliations:** 1 Sainsbury Wellcome Centre, University College London, London, UK; 2 Kavli Institute for Systems Neuroscience and Centre for Neural Computation, Norwegian University of Science and Technology, Trondheim, Norway; 3 Present Address: Inserm, CNRS, Institut du Cerveau - Paris Brain Institute - ICM, Sorbonne Université, Paris, France

**Keywords:** Image processing, Neural circuits, Software

## Abstract

High-resolution whole-brain microscopy provides a means for post hoc determination of the location of implanted devices and labelled cell populations that are necessary to interpret in vivo experiments designed to understand brain function. Here we have developed two plugins (brainreg and brainreg-segment) for the Python-based image viewer napari, to accurately map any object in a common coordinate space. We analysed the position of dye-labelled electrode tracks and two-photon imaged cell populations expressing fluorescent proteins. The precise location of probes and cells were physiologically interrogated and revealed accurate segmentation with near-cellular resolution.

## Introduction

Systems neuroscience often relies on the use of implanted devices and viral injections for the stimulation and recording of anatomically- or genetically-defined neuronal populations. To correctly interpret the resultant data, it is critical to map the location of the implanted devices or injections, and to pool results from multiple animals in a common anatomical coordinate system. Recent developments in microscopy and tissue clearing now allow for fully automated, high-resolution imaging of intact rodent brains^1^. There exist many methods for registering these 3D whole-brain microscopy datasets to an atlas, but these are often inflexible, time-consuming and require considerable computational skills^2^. Also, once registered there exist no open-source, user-friendly tools for the segmentation and analysis of any type of structure in these images. Here, we have therefore developed brainreg and brainreg-segment, two user-friendly tools that may be used to register and segment whole-brain microscopy datasets within minutes.

## Results

**Figure 1:**
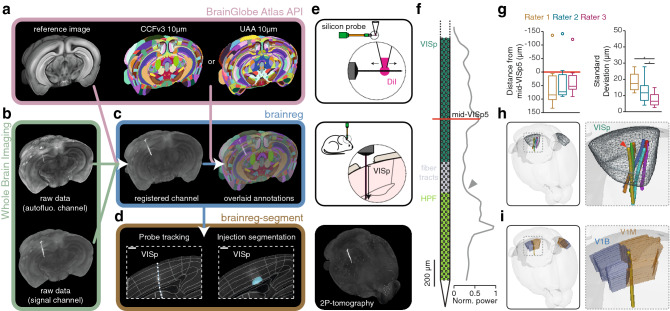
Overview of the brainreg/brainreg-segment workflow and validation of probe track tracing**. **(**a**) Atlas data including the reference anatomical image and the brain region annotations are sourced from the BrainGlobe Atlas API and used for image registration. Multiple atlases, such as the Allen Mouse Brain Common Coordinate Framework version 3 (CCFv3) and the Unified Anatomical Atlas (UAA) are supported. (**b**) Multichannel (e.g., labelled signal channel and a secondary autofluorescence channel) whole-brain microscopy data can be processed directly by brainreg without pre-processing (other than stitching). (**c**) Brainreg registers the sample data to the atlas reference image (and vice versa), allowing brain region annotations to be overlaid upon the raw data. (**d**) Brainreg-segment allows manual segmentation of labelled structures such as linear electrophysiological probes and fluorescent cell populations. Scale bars = 250 μm. (**e**) Schematic of the electrophysiological probe (Neuropixels) experiment. Top: A Neuropixels probe is coated with DiI by coming into contact with a suspended drop of the dye. Middle: The Neuropixels probe is inserted in the mouse brain above the primary visual cortical region (VISp) at a depth of 1750 or 2000 μm. Bottom: Projection of a whole-brain image (red channel) acquired with serial two-photon tomography, showing the DiI signal left by the Neuropixels probe. (**f**) Schematic of the probe and plot of the normalized Local Field Potential (LFP) power (500–1250 Hz) against depth. The red line shows the depth of the cortical LFP power peak, indicating the position of the middle of layer 5 (mid-VISp5). The grey arrowhead indicates the increase in LFP power when entering the hippocampal formation. (**g**) Left: Box plot showing the distance from landmark (mid-VISp5) for each rater (n = 7 per rater; positive distances are towards the pia). Right: Box plot showing the standard deviation of each rater (n = 7 per rater). (**h**) Rendering of the position of all the probe tracks (lines) within VISp (wireframe). Red arrowhead indicates the probe track shown in panel i. (**i**) One probe track (yellow) was identified as crossing the monocular primary cortex (V1M, brown) and the binocular primary cortex (V1B, blue) border.

Brainreg and brainreg-segment were developed as plugins for the napari^3^ multidimensional image viewer to leverage the existing scientific Python ecosystem for brain mapping, without requiring any computational knowledge. Using napari also allows other plugins to be used in combination with ours, such as those for reading of most microscopy image file formats. Brainreg allows drag-and-drop loading of raw data, and one-click registration to an array of brain atlases, provided by the BrainGlobe Atlas API^4^ and the algorithms underlying it have already been validated^5^. Brainreg loads the atlas (Fig. 1a) and the raw data (Fig. 1b), which are resampled and filtered prior to registration. The two images are aligned using affine, and then non-linear free-form deformation^6^. This process is entirely automated and requires no user input (for more details, see Ref.^5^). Using the resulting transformations, the atlas is overlaid upon the raw data, and the raw data is warped to the atlas (Fig. 1c). This allows the user to visualise and analyse data in a common coordinate space, as well as in the raw data space (for the analysis of individual brain structural morphology). Brainreg was developed to enable routine analysis on standard computing hardware. Registration of whole mouse-brain images to the Allen Mouse Brain Common Coordinate Framework version 3 (CCFv3, 25 μm resolution^7^) on a standard laptop (Dell XPS 9380) takes approximately five minutes (4 min 55 s ± 1 min 41 s, Supplementary Fig. 1). In addition to the standard structures provided by the atlas, brainreg-segment allows the user to manually annotate either 1D tracks, 2D areas, or 3D volumes (Fig. 1d). These segmented structures are analysed to determine their position and extent in the brain and can be exported to brainrender^8^ for visualisation in atlas space.

To demonstrate the robustness of our approach, we first assessed the reliability of brainreg itself. Three brains were registered (CCFv3, 25 μm resolution) 100 times each and the resulting registrations were compared at each iteration. We found that brainreg registrations were identical at each iteration for a given brain (voxel-to-voxel comparison, n = 3 brains) indicating that brainreg is consistent. We then proceeded to quantify the accuracy of the registration in real-life experimental conditions, by asking three expert neuroscientists to localise extracellular recording probes^9^ that had been coated with DiI and inserted in the primary visual cortex. After recordings, a total of 7 brains were imaged using serial two-photon tomography^10^ (Fig. 1e) and then processed with brainreg (CCFv3, 10 μm resolution). The experts were then asked to localise the silicon probe track using the brainreg-segment “Track tracing” function and the variability of track tracing was determined using the neocortical mid-layer 5 (mid-VISp5) as a “ground truth” landmark^11^ (Fig. 1f). The average distance from mid-VISp5 (all raters: median = 57.4 µm, range = − 141.7–134.1 µm, n = 21) was not different between raters and was always below 84.5 µm (rater 1: median = 84.5 µm, range = − 135.9–134.1 µm; rater 2: median = 73.1 µm, range = − 141.7–90.3 µm; rater 3: median = 52.8 µm, range = − 120.8–90.6 µm; *p* > 0.05 Wilcoxon signed rank test; Fig. 1g). Interestingly, the direction of error from the landmark was positive in 17/18 cases suggesting that there is a systematic underestimation of the probe track length (median = 69.4 µm, range = − 5.4–134.1 µm, n = 18 excluding outliers, see Methods; Fig. 1g). This likely reflects the difficulty of detecting the tip of the probe, resulting in experimenters tracing shorter tracks. We also found that while reliability differed between raters, it was always below an average of 17.4 µm SD (rater 1: median = 17.4 µm, range = 11.6–27.7 µm; rater 2: median = 11.7 µm, range = 4.1–27.8 µm; rater 3: median = 6.5 µm, range = 2.6–14.9 µm; *p* <  0.05 Wilcoxon signed rank test; Fig. 1g) and did not exceed 27.8 µm. These results show that, when compared to an electrophysiological “ground-truth” landmark (mid-VISp5), the accuracy of all raters was on average 69.4 µm. The accuracy and reliability were similar when using the 25 µm atlas, but not 50 µm or 100 µm (Supplementary Fig. 2). Since the probe tracks were analysed in the CCFv3 (Fig. 1h) and the Unified Anatomical Atlas^12^ (UAA, Fig. 1i) coordinate spaces, it was possible, for example, to track individual probes that traversed the boundaries of adjacent cortical areas.

**Figure 2:**
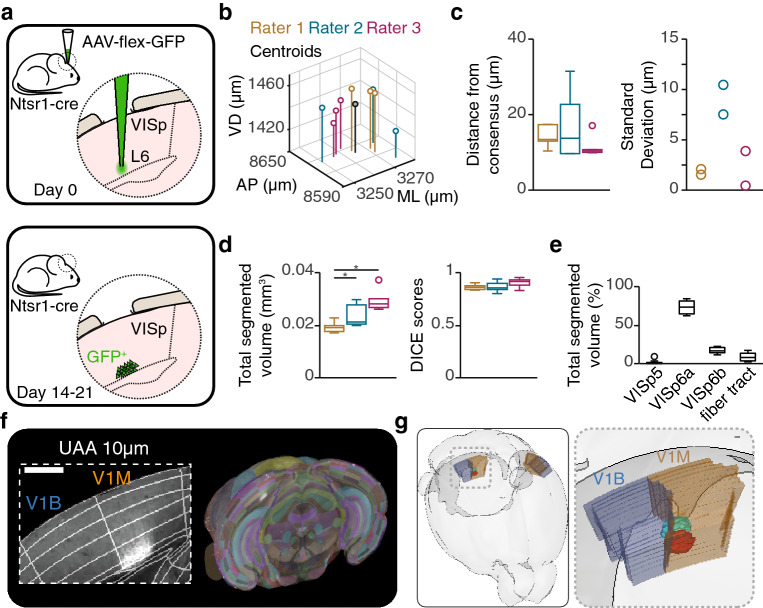
Segmentation of injection volumes using brainreg-segment. (**a**) Schematic of the injection segmentation experiment timeline. Top: Injection of an AAV carrying the fluorescent protein GFP in the primary visual cortex (VISp) of a Ntsr1-cre transgenic mouse at a depth of 900 μm (layer 6, L6). Bottom: Expression of GFP by a subset of cortical neurons (Ntsr1-positive cells). (**b**) Plot showing the 3D position of the segmented region centroids of each rater (open circles, n = 3 centroids per rater). The average centroid for all raters is also shown (grey circle). (**c**) Left: Box plot showing the distance from consensus for all centroids and for each rater. Right: Plot showing the standard deviation of each brain and for each rater. (**d**) Left: Box plot showing the total segmented volume for each rater. Right: Box plot showing the DICE scores for each rater. (**e**) Box plot showing the percentage of segmented volume in each CCFv3 brain region. (**f**) Left: coronal image of the primary visual cortex (inset) and projection of the first 850 brain images (green channel) acquired with serial two-photon tomography, overlaid with the UAA atlas. Scale bar = 500 μm. (**g**) Rendering of the segmented regions within the primary visual cortex.

A second important application of the brainreg/brainreg-segment pipeline is the segmentation of brain regions according to pathological irregularities, lesions or injections. We demonstrated this feature by asking 3 experts to manually segment a brain region where neurons expressed the green fluorescent protein (GFP) using the brainreg-segment “Region segmentation” function (Fig. 2a). In order to assess the performance of individual raters, each rater segmented each region 3 times. A centroid was computed for each segmented region and a “consensus” centroid was calculated by averaging all centroid positions (3 centroids per brain, 2 brains, n = 6; Fig. 2b). Then, each rater’s performance was assessed by calculating the Euclidean distance between their centroids and the “consensus” centroid. The average distances to consensus (median = 12.3 µm, range = 9.7–31.5 µm) were not significantly different between raters (rater 1: median = 13.4 µm, range = 10.4–17.4 µm; rater 2: median = 13.8 µm, range = 9.7–31.5 µm; rater 3: median = 10.4 µm, range = 9.9–17.1 µm; *p* > 0.05, Wilcoxon signed rank test; Fig. 2c). The distance to consensus did not exceed 31.5 µm. In addition, we assessed the intra-rater variability of region segmentation by computing the standard deviation of each rater. The standard deviation did not exceed 10.5 µm (Fig. 2c). The volumes reported by the 3 raters were all in the same order of magnitude, however volume estimation differed between raters (rater 1: median = 0.019 mm^3^, range = 0.017–0.022 mm^3^; rater 2: median = 0.021 mm^3^, range = 0.020–0.030 mm^3^; rater 3: median = 0.028 mm^3^, range = 0.026–0.037 mm^3^; *p* < 0.05, Wilcoxon signed rank test; Fig. 2d). Since these raters estimated a similar position for their respective brain segmentation centroids, the differences in volume reflect a difference in judgement of injection borders. The overlap of the brain segmentations was also explored by scoring each rater against a “consensus” segmented region (see Methods). The DICE scores were not significantly different between raters (rater 1: median = 0.86, range = 0.83–0.90; rater 2: median = 0.92, range = 0.83–0.95; rater 3: median = 0.85, range = 0.80–0.94; *p* > 0.05, Wilcoxon signed rank test; Fig. 2d) and did not fall below 0.80, which indicates a high degree of consensus between raters. The advantage of brainreg-segment was further demonstrated using two extra features. Firstly, the brain segmentation was subdivided to reflect its spread through different brain regions (Fig. 2e). Secondly, we used the UAA to quantify the spread of the injection relative to the monocular and binocular primary visual cortex border (V1M/V1B; Fig. 2f and g).

**Figure 3:**
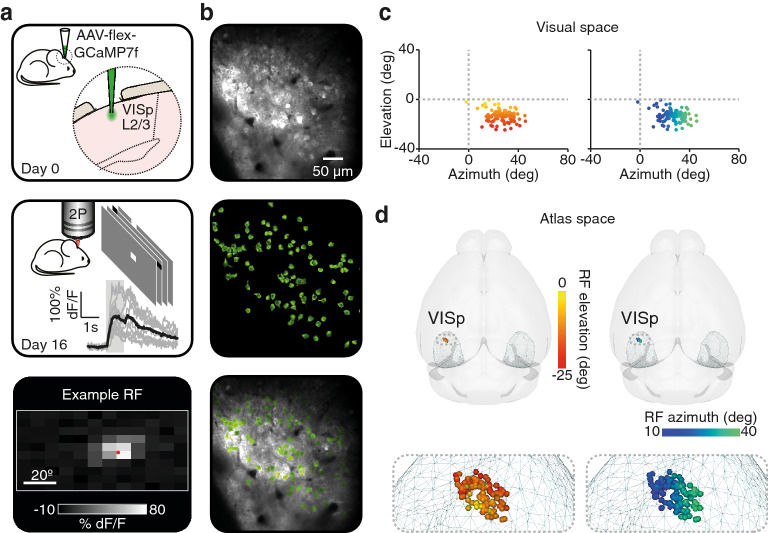
Localization of recorded cell populations according to the centroid of the labeled volume**. **(**a**) Schematic of the functional imaging experiment timeline. Top: Injection of an AAV carrying the fluorescent indicator GCaMP7f in the primary visual cortex (VISp) at a depth of 250 μm (layer 2/3, L2/3). Middle: Two-Photon calcium imaging of L2/3 neurons in awake head-fixed mice, while presenting sparse noise stimuli. Example recorded trials from one neuron are shown in grey and their average response in black, aligned to the onset of stimulus (shaded grey area). Bottom: Average Receptive Field (RF) from one neuron, shown in visual space. (**b**) Top: two-photon grayscale image of GCaMP7f-expressing cells. Middle: Regions Of Interest (ROIs, green) determined by automated detection of labeled cells using suite2p. Bottom: Overlay of the Two-Photon image (grayscale) and the ROIs (green). (**c**) Left: centroids of all RFs recorded in one animal, color-coded according to their elevation in visual space. Right: centroids of all RFs recorded in one animal, color-coded according to their azimuth position in visual space. (**d**) Left: rendering of the position in atlas space of all recorded cells shown in panel c, color-coded according to their elevation in visual space. Right: rendering of the position in atlas space of all recorded cells shown in panel c, color-coded according to their azimuth position in visual space.

To highlight the potential application of this approach, we also performed in vivo calcium imaging to determine the functional and anatomical topography of labelled layer 2/3 visual cortical neurons. GCaMP7f-positive cells were imaged under a two-photon microscope and their individual receptive fields (RFs) were mapped (Fig. 3a and b). The centroid of each RF was computed in visual space and color-coded according to their elevation/azimuth position (Fig. 3c). The location of each functionally imaged cell was extracted and positioned in atlas space relative to the centroid of the GCaMP7f injection site, which was computed using the brainreg-segment “Region segmentation” function, demonstrating the retinotopic organisation of V1 RFs (Fig. 3d).

## Discussion

To our knowledge, there has been only a single report of this type of analysis using whole-brain microscopy. Liu et al. (2020)^13^ used light sheet microscopy to image electrophysiological probes and a semi-automated approach to align the images to an atlas (CCFv3). However, while the accuracy of their approach is comparable to that reported here, it is specific to linear electrophysiological probes and the software is not publicly available. One of the main advantages of our approach is its myriad of applications and its flexibility since it is designed to allow segmentation of multiple structures of any size and shape. These tools can be used to analyse the location of brain structures and fibre tracts, pathologically- or experimentally-induced lesions or recording/stimulation devices such as optical fibres (Supplementary Fig. 3) or miniscopes^14^. Since they are built upon open-source Python data analysis and visualisation tools including the BrainGlobe Atlas API^4^ they are compatible with multiple existing, as well as future brain atlases. Because the tools in the BrainGlobe suite are compatible with these atlases, the results of brainreg-segment can be directly compared with results from other software, for example cellfinder, to compare the distribution of individually labelled cells to a bulk injection site in physiological conditions^15^ or after experimentally-induced trauma^16^. Also, these tools were developed as plugins for napari to provide interoperability with other analysis software, and importantly to streamline installation and use. The bundled napari application allows use without installing Python and the plugins can be installed directly from the graphical user interface, making these tools accessible to the wider neuroscience community. Here, we have chosen a manual approach to segmentation as it is relatively fast—segmentation times depend on the structure being segmented, but are typically from seconds to a few (e.g., five) minutes. A manual approach was chosen for maximal flexibility, to allow the segmentation of any type of structure characterised by different signal properties (e.g., fluorescent expression or absence of tissue). It also requires no optimisation, and it is relatively consistent between raters. However, as whole-brain microscopy becomes easier and cheaper, and large multi-laboratory efforts become more common in neuroscience (e.g., see Ref.^17^), fully automated methods^18, 19^ could provide additional refinements.

## Methods

### Surgical procedures

All experiments were performed on 9–18 week old C57BL/6, B6.FVB(Cg)-Tg(Ntsr1-cre)Gn220Gsat/Mmucd (Ntsr1-cre), B6.Cg-GT(ROSA)26 Sortm14(CAG-tdTomato)Hze/J crossed to Gad2tm2(cre)Zjh/J or B6.FVB(Cg)-Tg(Adora2a-cre)KG139Gsat/Mmucd male mice in accordance with the UK Home Office regulations (Animal (Scientific Procedures) Act 1986), approved by the Animal Welfare and Ethical Review Body (AWERB; Sainsbury Wellcome Centre for Neural Circuits and Behaviour) and in compliance with ARRIVE guidelines. All surgical procedures, including the implantation of head plates, craniotomies, cranial windows and virus injections, were carried out under isoflurane (2%–5%) and after carprofen (5 mg/kg, s.c.) had been administered.

### Extracellular recordings

For extracellular recordings, C57BL/6 mice (10–18 weeks old) were used. Mice were anesthetized under isoflurane (2%–5%), implanted with a head plate and allowed to recover for 48 h. Then, animals were anesthetized under isoflurane (2%–5%) and a small craniotomy (1 × 1 mm) was drilled over the primary visual cortex. The craniotomy was sealed with silicon (kwik-cast) and the animals were allowed to recover for 2 h. The head was then positioned with a pitch angle of 30 degrees (nose down). The silicon probe (IMEC, Belgium) was first coated with DiI (Molecular Probes, Thermo Fisher Scientific, USA). The probe was then positioned perpendicular to the brain surface and the tip inserted to 1750 or 2000 µm from the pial surface. The silicon probe data was acquired using an FPGA card (KC705, Xilinx, USA) and SpikeGLX (https://github.com/billkarsh/SpikeGLX) and filtered at 300 Hz.

To determine the middle of layer 5 electrophysiologically, we used the depth profile of the high-frequency LFP power^11^. Briefly, for each non-reference channel on the probe, the average power in the frequency range 500–1250 Hz was computed (MATLAB, The MathWorks Inc., bandpower function). Reference channels were interpolated (MATLAB interp1), and the depth profile was smoothed across 10 channels (MATLAB smooth, which implements a moving average) to reduce variability across channels. The location, in the cortex, of the peak in this depth profile was chosen as the middle of layer 5 (mid-VISp5). The increase in LFP power when entering the hippocampal formation was used as an additional landmark to confirm the correct depth.

### Virus injections

For virus injections, the same procedure was followed as described in Ref.^20^. Briefly, Ntsr1-cre mice (10–15 weeks old) were anaesthetized under isoflurane (2%–5%) and craniotomies performed. Cre-dependent AAV viruses encoding the fluorescent protein GFP were injected in layer 6 of the primary visual cortex (at a depth of 900 µm). After injections, the craniotomy was sealed with silicon (kwik-cast) and animals were allowed to recover for 2–3 weeks. Viruses were delivered at a rate of 1–2 nl/s using Nanoject III (Drummond Scientific, USA).

For in vivo GCaMP7f imaging, mice were anaesthetized with isoflurane (2%–5%) and implanted with a headplate. A craniotomy (3 mm) was drilled over the primary visual cortex, and 100 nl of a virus encoding GCaMP7f (AAV1-syn-jGCaMP7f-WPRE, titre: 4.75 × 10^12^ ml^−1^) was injected in layer 2/3 of the primary visual cortex (Bregma: − 2.7 mm, ML: − 2.58 mm, depth: 0.25 mm) at a rate of 1 nl/s. The craniotomy was sealed by implanting a 3 mm diameter round glass coverslip (#1, Harvard Apparatus, USA) to create a chronic cranial window.

### Two-photon calcium imaging

Prior to imaging experiments, mice were handled and accustomed to head fixation for 3 days. GCaMP7f fluorescence was imaged 16 days after viral injection in awake head-fixed mice using a two-photon moveable objective microscope (Sutter Instruments, USA) coupled to a Ti–Sapphire laser (Insight Deepsee, Spectra Physics, USA) tuned to 930 nm. Neurons located ~ 200 µm below the surface of the brain were visualised with a 16 × 0.8 NA water-immersion objective (Nikon, Japan) and GaAsP PMTs (Hamamatsu Photonics, Japan) in a 390 × 390 µm^2^ field of view and images (512 × 512 pixels) were acquired with a resonant scanner (8 kHz, Cambridge Technology) at a frame rate of 30 Hz. To minimize photodamage, the excitation laser power was adjusted to the lowest intensity required for the imaging depth.

### Visual stimulation

Visual stimuli for receptive field (RF) mapping were generated in MATLAB using the Psychophysics Toolbox^21^ and presented on an LCD monitor (Samsung C24FG73FQU, 120 Hz refresh rate) positioned 20 cm from the right eye at approximately 45° to the long axis of the animal, such that it covered ∼105° (azimuth) × 75° (elevation) of visual space. Stimuli consisted of black and white squares on a grey background, presented one at a time (1 Hz) in a randomised order, at one of 120 positions (12 × 10 matrix) covering the full surface of the monitor. Each position was repeated 10 times. The backlight of the monitor was synchronized to one turn-around point of the resonant scanner (where data is not acquired), so that the monitor flickered at 8 kHz and light from the screen is only on in between the scanning of two subsequent lines. A photodiode was positioned over the upper-left corner of the monitor and data was acquired in parallel with imaging to provide an accurate stimulus timestamp.

### Receptive field analysis

Pre-processing of all raw calcium movie data including image registration and automated cell detection was performed with suite2p^22^. All subsequent analysis was performed with custom scripts written in MATLAB. Neuropil subtraction to correct for contamination of the ROI calcium traces by surrounding neuropil and calculation of ΔF/F was computed as described in Ref.^23^. The response to each individual patch was taken as the mean dF/F during the 1 s stimulus presentation minus the mean dF/F during the previous 1 s (baseline). In the following, responses to black and white stimuli were analysed separately. To assess whether ROIs had a significant RF, responses to each patch position were grouped and a Kruskal–Wallis test was performed (*p* < 0.05). Only ROIs with significant RFs were subsequently analysed. To compute the RF centroid, the median response to each stimulus position was taken to create a raw 12 × 10 RF map. These RF maps were smoothed (imgaussfilt) and upsampled by a factor of 10 (imresize). Upsampled RFs were then thresholded (*threshold* = max(RF)–*std*(RF)) and the centroid of the largest subdomain after thresholding was taken as the RF centroid.

### Optical fiber implantation

For implantation of fiber optic cannulae (Doric Lenses 0.57NA, 400 μm diameter), 9–15 weeks old mice were used. Mice were anesthetized under isoflurane (2%-5%), a small craniotomy (1 × 1 mm) was drilled directly above the tail of the striatum, the dura was removed and implants were inserted. Implants were attached to the skull using UV cure dental cement (RelyX Unicem 2 Automix) reinforced with dental cement (Super-Bond). Following surgery, the mouse was returned to its home-cage to recover.

### Serial two-photon imaging

Following silicon probe recordings, virus injections or optical fiber implantations, animals were deeply anaesthetized and transcardially perfused with cold phosphate buffer (PB, 0.1 M) followed by 4% paraformaldehyde (PFA) in PB (0.1 M) and brains left overnight in 4% PFA at 4 °C. Brains were then embedded in 4% agar and imaged using serial two-photon tomography^10^, using a commercially available system as previously described^20^ or a custom system controlled by ScanImage (v5.6, Vidrio Technologies, USA) using BakingTray^24^. Images were acquired as tiles, and stitched using a custom FIJI^25^ plugin (modified from Ref.^26^) or StitchIt^27^. For silicon probe tracking, images were acquired with 20 μm axial sampling with 4.6 × 4.6 μm pixels. For GFP-injection mapping, images were acquired with 1 × 1 μm pixels and 5 μm axial sampling, before downsampling to 5 μm isotropic sampling. For optical fiber localisation, images were acquired with 2.23 × 2.23 pixels and 5 μm axial sampling, before downsampling to 5 μm isotropic sampling. For GCaMP7f-injection mapping, images were acquired with 2.23 × 2.23 μm pixels and 10 μm axial sampling. All images were saved as a series of 2D TIFF files.

### Image registration

Images were registered to an atlas, either the Allen Mouse Brain Common Coordinate Framework version 3^7^ or the Unified Anatomical Atlas^12^.⁠ All atlas data was provided by the BrainGlobe Atlas API^4^. The command line tool expects TIFF files (either 3D or a series of 2D images), but the napari plugin is compatible with most microscopy image file formats by leveraging the rich ecosystem of existing reader plugins (listed at https://www.napari-hub.org/). For registration, the sample image data was initially downsampled to the voxel spacing of the atlas used and reoriented to align with the atlas orientation using bg-space^28^. The 10 μm atlas was used for the electrophysiological probe experiments, and 25 μm for the injection mapping. The downsampled image and the atlas reference images were then filtered using scikit-image^29^ and SciPy^30^⁠, in order to remove-high frequency noise (greyscale opening and flat-field correction). Registration was performed in two steps, firstly images were linearly aligned using a block-matching approach^31^ using the NiftyReg reg_aladin command. Non-linear registration was then performed with the NiftyReg reg_f3d implementation of the free-form deformation algorithm^6^. This approach overlays a grid of reference points onto the image to be transformed, these are then moved to locally warp the image. Image similarity (i.e., registration accuracy) was optimised by calculating the normalized mutual information. For more details, see Ref.^5^. The transformation from atlas space to sample space was then applied to the atlas annotations image (and an atlas hemispheres image) so that brain region annotations could be overlaid on the image. For analysis in atlas space, the affine transform was inverted (using reg_transform), and the non-linear transformation was performed in reverse (from sample to atlas).

### Manual segmentation and region analysis

To manually segment structures within the brain, a graphical user interface was developed using Qt (www.qt.io/ via qtpy https://github.com/spyder-ide/qtpy) and napari^3^. This tool (brainreg-segment) allows 1, 2, and 3-dimensional structures to be segmented, analysed (e.g., distribution within brain regions) and summarised (e.g., centroids or tissue volumes).

A full user-guide for brainreg-segment is available at https://docs.brainglobe.info/brainreg-segment, but a summary of the analysis process is as follows. Upon starting the plugin, the user is presented with an option to load data previously registered with brainreg. The user can load the entire brainreg output directory, which loads the appropriate atlas data and image metadata. The analysis can be carried out in the coordinate space of the atlas, the sample, or alternatively, the atlas alone can be loaded. All analysis in this manuscript was performed in atlas space.

For segmentation of 1D structures (e.g., electrophysiological probes), there is a “Track tracing” button which loads a new panel. Selecting the “Add track” button allows the user to follow the path of the structure by adding points along its length. These points can then be fitted (“Trace tracks” button) using spline interpolation and the user can choose the order of the spline fit, a smoothing factor (how closely or not to fit the points) and the number of points along the track to analyse. In this manuscript, we used a linear fit (order 1), smoothing of 0.1 and 1750 or 2000 points, which corresponds to one sampling point per micron. The analysis of this fit results in a CSV file detailing the brain region at each of the sampling points along the length of the structure. The outliers in Fig. 1g all originate from one single brain and each rater has it as an outlier. Taking a closer view to this particular brain showed that the quality of the DiI labelling was suboptimal (due to brain surface deformation), indicating that the quality of the DiI tracing and brain tissue have a strong effect on track tracing accuracy and that errors introduced by suboptimal labelling are systematically reported by all raters.

For segmentation of 2/3D structures (e.g., injection sites), there is a “Region segmentation” button which loads a different panel. Selecting the “Add track button” allows the user to segment the structure by “painting” onto the image in successive 2D planes. The spatial distribution of the structure can then be analysed (“Analyse regions” button), and various parameters can be saved, such as the volume of the structure in each brain region, and summary statistics such as the centroid of each volume.

There is no limit to the number of structures that can be segmented using this tool, and there is a button to save the structures (to be reloaded at a later date). If analysis is performed in atlas space, the structures can be directly exported in a brainrender^8^ compatible format (.npy or .obj) for visualisation in 3D.

### Computing hardware

For timing of registration, analyses were performed on three machines, a desktop workstation (HP Z8 G4), a high-specification laptop (3XS Deep Learning GO) and a “standard” laptop (Dell XPS 9380). The workstation ran Ubuntu 16.04, and had 384 GB RAM and dual Intel Xeon 6132 14 core (28 thread) CPUs, but brainreg was limited to 6 cores (12 threads) for registration. The high-specification laptop ran Ubuntu 18.04 and had 64 GB RAM, and a single Intel i9-9900 K (8 core, 16 thread) CPU. The “standard” laptop ran Ubuntu 18.04 and had 16 GB RAM, and a single Intel i7-8565U (4 core, 8 thread) CPU. In all cases, data were stored on an internal or external solid-state drive.

### Data analysis and visualisation

To compare segmented injection variability, a consensus injection image was calculated from all nine (three repeats by three experts) manual segmentations. Voxels which were found in over half of the segmentations (i.e. at least five) made up the binary mask of the consensus image. Individual expert segmentations were then compared to this consensus by calculating the DICE^32^ score.

Plots were generated using MATLAB (The MathWorks Inc.) and image visualisation was performed using napari and brainrender.

## Supplementary Information

Supplementary Information.

## Data Availability

The software outlined in this manuscript is available as part of the BrainGlobe suite of computational neuroanatomy tools. The software is open source, written in Python 3 and runs on standard desktop computing hardware. Brainreg is fully supported on Linux and Windows, and brainreg-segment on Linux, Windows and macOS. Source code is available at https://github.com/brainglobe/brainreg, https://github.com/brainglobe/brainreg-napari and https://github.com/brainglobe/brainreg-segment. Documentation and tutorials are available at https://docs.brainglobe.info.

## References

[1] Osten P, Margrie TW (2013). Mapping brain circuitry with a light microscope. Nat. Methods.

[2] Tyson, A. L. & Margrie, T. W. Mesoscale microscopy and image analysis tools for understanding the brain. Progress in Biophysics and Molecular Biology. *In press* (2021) 10.1016/j.pbiomolbio.2021.06.013 PMC8786668 34216639

[3] Sofroniew, N., Lambert, T., Evans, K., Nunez-Iglesias, J., Winston, P., Bokota, G., Yamauchi, K., Solak, A. C., ziyangczi, Peña-Castellanos, G., Bussonnier, M., Buckley, G. & Pop, D. D.* et al.* (2021). napari/napari: 0.4.8.

[4] Claudi F, Petrucco L, Tyson AL, Branco T, Margrie TW, Portugues R (2020). BrainGlobe Atlas API: a common interface for neuroanatomical atlases. J. Open Source Softw..

[5] Niedworok CJ, Brown APY, Jorge Cardoso M, Osten P, Ourselin S, Modat M, Margrie TW (2016). AMAP is a validated pipeline for registration and segmentation of high-resolution mouse brain data. Nat. Commun..

[6] Modat M, Ridgway GR, Taylor ZA, Lehmann M, Barnes J, Hawkes DJ, Fox NC, Ourselin S (2010). Fast free-form deformation using graphics processing units. Comput. Methods Programs Biomed..

[7] Wang Q, Ding SL, Li Y, Royall J, Feng D, Lesnar P, Graddis N, Naeemi M, Facer B, Ho A, Dolbeare T, Blanchard B, Dee N, Wakeman W, Hirokawa KE, Szafer A, Sunkin SM, Oh SW, Bernard A, Phillips JW, Hawrylycz M, Koch C, Zeng H, Harris JA, Ng L (2020). The Allen mouse brain common coordinate framework: a 3D reference atlas. Cell.

[8] Claudi F, Tyson AL, Petrucco L, Margrie TW, Portugues R, Branco T (2021). Visualizing anatomically registered data with brainrender. Elife.

[9] Jun JJ, Steinmetz NA, Siegle JH, Denman DJ, Bauza M, Barbarits B, Lee AK, Anastassiou CA, Andrei A, Aydin Ç, Barbic M, Blanche TJ, Bonin V, Couto J, Dutta B, Gratiy SL, Gutnisky DA, Häusser M, Karsh B (2017). Fully integrated silicon probes for high-density recording of neural activity. Nature.

[10] Ragan T, Kadiri LR, Venkataraju KU, Bahlmann K, Sutin J, Taranda J, Arganda-Carreras I, Kim Y, Seung HS, Osten P (2012). Serial two-photon tomography for automated ex vivo mouse brain imaging. Nat. Methods.

[11] Senzai Y, Fernandez-Ruiz A, Buzsáki G (2019). Layer-specific physiological features and interlaminar interactions in the primary visual cortex of the mouse. Neuron.

[12] Chon, U., Vanselow, D. J., Cheng, K. C. & Kim, Y. Enhanced and unified anatomical labeling for a common mouse brain atlas. *Nat. Commun.* **10**, 5067 (2019). 10.1038/s41467-019-13057-w PMC6838086 31699990

[13] Liu LD, Chen S, Hou H, West SJ, Faulkner M, The International Brain Laboratory, Economo MN, Li N, Svoboda K (2021). Accurate localization of linear probe electrode arrays across multiple brains. eNeuro 8 (6).

[14] Obenhaus HA, Zong W, Jacobsen RI, Rose T, Donato F, Chen L, Cheng H, Bonhoeffer T, Moser M-B, Moser EI (2021). Functional network topography of the medial entorhinal cortex. BioRxiv.

[15] Tyson AL, Rousseau CV, Niedworok CJ, Keshavarzi S, Tsitoura C, Cossell L, Strom M, Margrie TW (2021). A deep learning algorithm for 3D cell detection in whole mouse brain image datasets. PLoS Comput. Bio..

[16] Wang Z, Romanski A, Mehra V, Wang Y, Campbell BC, Petsko GA, Tsoulfas P, Blackmore M (2021). Brain-wide quantification of the supraspinal connectome. BioRxiv.

[17] Abbott LF, Angelaki DE, Carandini M, Churchland AK, Dan Y, Dayan P, Deneve S, Fiete I, Ganguli S, Harris KD, Häusser M, Hofer S, Latham PE, Mainen ZF, Mrsic-Flogel T, Paninski L, Pillow JW, Pouget A, Svoboda K, Witten IB, Zador AM (2017). an international laboratory for systems and computational neuroscience. Neuron.

[18] Breiman L (2001). Random forests. Mach. Learn..

[19] Ronneberger O, Fischer P, Brox T, Navab N, Hornegger J, Wells W, Frangi A (2015). U-Net: convolutional networks for biomedical image segmentation. Medical image computing and computer-assisted intervention – MICCAI 2015. MICCAI 2015. Lecture Notes in Computer Science.

[20] Vélez-Fort M, Rousseau CV, Niedworok CJ, Wickersham IR, Rancz EA, Brown APY, Strom M, Margrie TW (2014). The stimulus selectivity and connectivity of layer six principal cells reveals cortical microcircuits underlying visual processing. Neuron.

[21] Brainard DH (1997). The psychophysics toolbox. Spat. Vis..

[22] Pachitariu M, Stringer C, Dipoppa M, Schröder S, Rossi LF, Dalgleish H, Carandini M, Harris KD (2017). Suite2p: beyond 10,000 neurons with standard two-photon microscopy. BioRxiv.

[23] de Vries SEJ, Lecoq JA, Buice MA, Groblewski PA, Ocker GK, Oliver M, Feng D, Cain N, Ledochowitsch P, Millman D, Roll K, Garrett M, Keenan T, Kuan L, Mihalas S, Olsen S, Thompson C, Wakeman W, Waters J (2020). A large-scale standardized physiological survey reveals functional organization of the mouse visual cortex. Nat. Neurosci..

[24] Campbell, R. A. A., BakingTray: Serial-section automated anatomy extension for ScanImage. (2020a) 10.5281/zenodo.3631610

[25] Schindelin J, Arganda-Carreras I, Frise E, Kaynig V, Longair M, Pietzsch T, Preibisch S, Rueden C, Saalfeld S, Schmid B, Tinevez J-Y, White DJ, Hartenstein V, Eliceiri K, Tomancak P, Cardona A (2012). Fiji: an open-source platform for biological-image analysis. Nat. Methods.

[26] Preibisch S, Saalfeld S, Tomancak P (2009). Globally optimal stitching of tiled 3D microscopic image acquisitions. Bioinformatics.

[27] Campbell R. A. A., Blot, A., lguerard., (2020b). StitchIt: Stitching of large tiled datasets. 10.5281/zenodo.3941901

[28] Petrucco, L. & Tyson, A. L. bg-space (2021).10.5281/zenodo.4552537

[29] Van Der Walt S, Schönberger JL, Nunez-Iglesias J, Boulogne F, Warner JD, Yager N, Gouillart E, Yu T (2014). Scikit-image: image processing in python. PeerJ.

[30] Virtanen P, Gommers R, Oliphant TE, Haberland M, Reddy T, Cournapeau D, Burovski E, Peterson P, Weckesser W, Bright J, van der Walt SJ, Brett M, Wilson J, Millman KJ, Mayorov N, Nelson ARJ, Jones E, Kern R, Larson E (2020). SciPy 1.0: fundamental algorithms for scientific computing in Python. Nat. Methods.

[31] Ourselin S, Roche A, Subsol G, Pennec X, Ayache N (2001). Reconstructing a 3D structure from serial histological sections. Image Vis. Comput..

[32] Dice LR (1945). Measures of the amount of ecologic association between species. Ecology.

