# Supplementary material for: Accurate determination of marker location within whole-brain microscopy images
Source: Sci Rep. 2022 Jan 18;12:867. doi: 10.1038/s41598-021-04676-9 (PMC8766598; doi:10.1038/s41598-021-04676-9)
Supplement: Supplementary file 1 — Supplementary Information. [file 41598_2021_4676_MOESM1_ESM.pdf]

Supplementary results

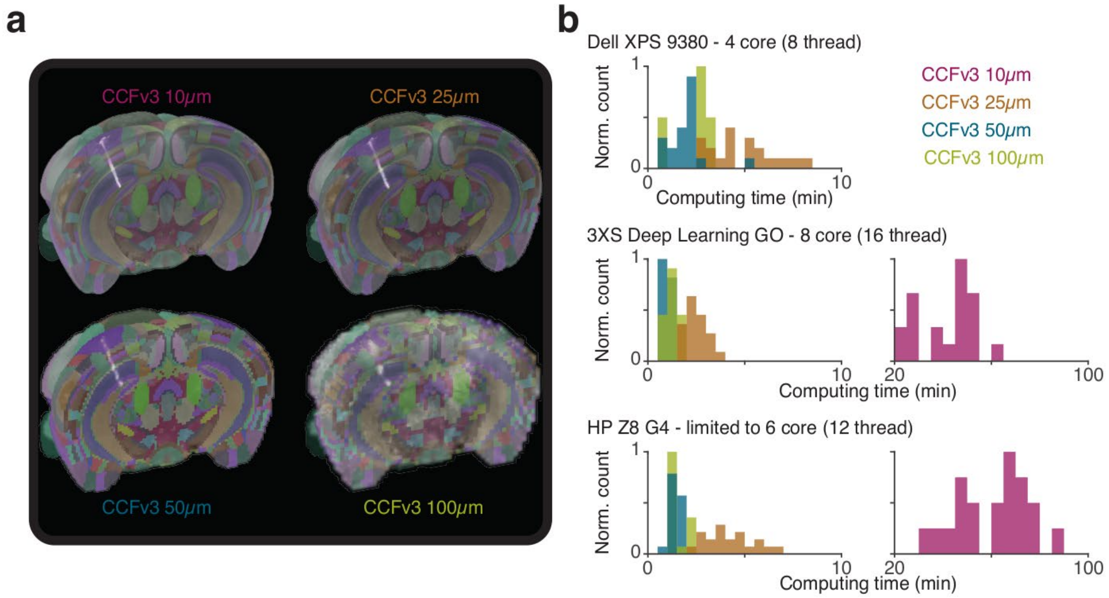

Supplementary Figure 1: Brainreg tested with different atlas resolutions and computing hardware.
a, Registration of a single brain to the CCFv3 at 10, 25, 50 and 100 µm isotropic resolutions. **b,** Top: Registration at 25, 50 and 100 µm isotropic resolutions on a standard laptop. Middle: Registration at 10, 25, 50 and 100 µm isotropic resolutions on a high-specification laptop. Bottom: Registration at 10, 25, 50 and 100 µm isotropic resolutions on a desktop workstation. Registration timings: 10 µm - high-specification laptop: 41min 15sec ± 11min 39sec, workstation: 60min 18sec ± 15min 30sec ; 25 µm - standard laptop: 4min 55sec ± 1min 41sec, high-specification laptop: 2min 33sec ± 35sec, workstation: 4min 14sec ± 1min 14sec.

Brainreg retrieves atlas data from the BrainGlobe Atlas API, which means that in addition to using different brain region annotations, it can also be used to register data at different resolutions. Some atlases, such as the CCFv3, are available at different resolutions (10, 25, 50 and 100 µm isotropic resolutions, Supplementary Fig. 1a). Registration using high-resolution atlases can take a long time, and require specific computing resources (e.g., sufficient memory). To test the computational hardware required by brainreg, the registration speed was tested on two datasets using the CCFv3 five times at each resolution. Firstly, a standard laptop was tested. The 10 µm isotropic resolution atlas could not be used, as the memory required (~30GB) was not available, but registration using the other atlases was fast (e.g., 25 µm = 4min 55sec ± 1min 41sec; Supplementary Fig. 1b). We also used a high-specification laptop and a desktop image analysis workstation. Registration using the 10 µm isotropic resolution atlas took around an hour (laptop: 41min 15sec ± 11min 39sec, workstation: 60min 18sec ± 15min 30sec), but all other resolutions took less time (e.g., 25 µm: laptop = 2min 33sec ± 35sec, workstation = 4min 14sec ± 1min 14sec; Supplementary Fig. 1b).

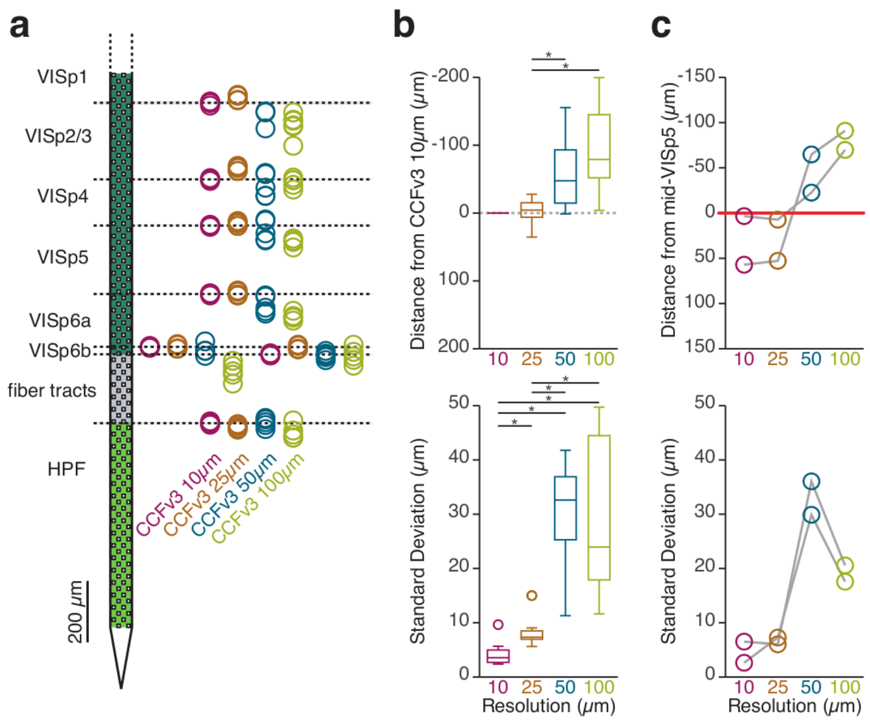

Supplementary Figure 2: Silicon probe track tracing at multiple resolutions with brainreg-segment.
a, Schematic of the probe and the position of each cortical layer crossing extracted from each CCFv3 atlas resolution (circles, n = 5 track tracings per atlas). Plot of the average layer crossing depth for the CCFv3 10 μm atlas (dashed black line) and standard deviation (very thin, shaded grey line). **b,** Top: Box plot showing the layer crossing depth distance from the CCFv3 10 μm atlas layer crossing (dashed grey line). Bottom: Box plot showing the standard deviation for each atlas. **c,** Top: Plot showing the distance from landmark (mid-VISp5, red line) for each brain and each atlas. Bottom: Plot showing the standard deviation for each brain and each atlas.

The accuracy and reliability of localising electrophysiological probe tracks using brainreg/brainreg-segment was studied further by testing different atlas resolutions. Rater number 3 was asked to trace probe tracks in 2 brains registered at 4 resolutions (CCFv3 at 10, 25, 50 and 100 μm isotropic resolutions). Each track was traced 5 times, to assess the rater's reliability (Supplementary Fig. 2a). The average distance of layer crossings was then compared to the layer crossing depths acquired by the same rater in the 10 μm resolution brain (2 brains, 7 cortical layer crossings; n = 14 per atlas). The distances recorded in the 25 μm resolution brains spread closely to the average layer crossings recorded in the 10 μm brains (median = -4.3 μm, range = -27.8 to 35.4 μm; Supplementary Fig. 2b). However, the distances recorded in the 50 μm and 100 μm resolution brains were significantly greater (50 μm atlas: median = -47.5 μm, range = -155.4 to +1.2 μm; 100 μm atlas: median = -79.0 μm, range = -199.6 to -4.0 μm, respectively; $p<0.05$, Wilcoxon rank sum test). In fact, in both brains recorded at a resolution of 50 or 100 μm resolution, the localisation of layer 1 (VISp1) and layer 6b (VISp6b), the thinnest of all cortical layers, were missed in several tracings. Interestingly, the accuracy deteriorated in one direction, indicating that lowering the brain resolution resulted in overestimating the probe length. Finally, the standard deviation of rater 3 (5 traces per brain, 2 brains, 7 cortical layer crossings; n = 14 SDs per atlas; Supplementary Fig. 2b) were significantly different, but of the same order of magnitude for brains recorded at 10 or 25 μm (10 μm atlas: median = 3.5 μm, range = 2.4 to

9.6 μm; 25 μm atlas: median = 7.3 μm, range = 5.6 to 15.0 μm; $p<0.05$, Wilcoxon signed rank test). However, the SDs significantly increased in brains recorded at 50 and 100 μm (50 μm atlas: median = 32.6 μm, range = 11.3 to 41.8 μm; 100 μm atlas: median = 23.9 μm, range = 11.6 to 49.7 μm; $p<0.05$, Wilcoxon rank sum test), indicating that the reliability of track tracing deteriorated with lower resolutions. Similar results were found when comparing the distance to the "ground-truth" (mid-VISp5) landmark: the tracing accuracy and reliability was similar between brains recorded at 10 and 25 μm, while they deteriorated when tracing in brains at resolutions of 50 and 100 μm (Supplementary Fig. 2c). Overall, these results show that using the brain resolution of 25 μm has little effect in the localisation of probe tracks, while at the same time, takes full advantage of processing speed.

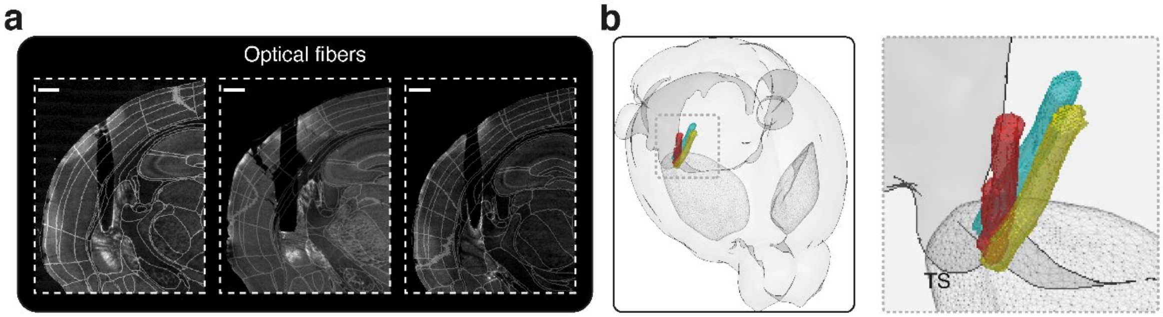

Supplementary Figure 3: Registration of brains with optical fibre tracts
a, Coronal sections of the fibre implantation sites for 3 different brains, with the registered outlines from the Allen atlas overlaid in sample space (CCFv3, 10 μm resolution). **b**, Left: Rendering of the whole brain and caudoputamen (wireframe), with each of the three fiber track volumes (red, blue and yellow) shown in atlas space. Right: A close up view of the three tracks and the tail of the striatum (TS) in atlas space. Scale bar = 500 μm.

To test the robustness of brainreg, we performed registration in brains characterised by a discontinuity of tissue structure left by the insertion of 400 μm optical fibres into the brain. We did not observe any significant registration errors caused by the tissue damage left by the optical fibers (Supplementary Fig. 3a). We then used brainreg-segment to analyse the position of the fiber tracks and confirmed the location of their tips, targeting the tail of the striatum (Supplementary Fig. 3b).
